# Suicidal Behavior in Nepali motion pictures of three decades (1990–2020): A content analysis

**DOI:** 10.1002/brb3.3366

**Published:** 2023-12-31

**Authors:** Rakesh Singh, Sharika Mahato, Kritika Singh, Niranjan Raman Puri, Susma Baniya, SM Yasir Arafat

**Affiliations:** ^1^ Research Department Transcultural Psychosocial Organization Nepal Kathmandu Nepal; ^2^ Monitoring, Evaluation and Research Department Plan International Nepal Lalitpur Nepal; ^3^ Department of Public Health National Open College Lalitpur Nepal; ^4^ Emergency Department Patan Academy of Health Sciences Lalitpur Nepal; ^5^ Department of Psychiatry Enam Medical College and Hospital Dhaka Bangladesh

**Keywords:** Nepal, Nepali movie, suicidal behavior, suicide

## Abstract

**Background:**

Assessment of the depiction of suicidal behavior in motion pictures would reveal the social representation of suicide that would foster suicide prevention in a country.

**Objectives:**

We aimed to assess how suicidality has been depicted in Nepali movies by scrutinizing their contents against the sociodemographic checklist and WHO media guidelines for suicidal reporting.

**Methods:**

This is a narrative quantitative analysis of suicidal behavior portrayals in the Nepali motion pictures that are publicly and freely accessible.

**Results:**

Overall, out of the 573 scrutinized movies, we found ten movies consisting of 11 characters (i.e., the prevalence is 1.75%) showing suicidal behavior. The majority of suicidal behavior was seen in males 6 (54.5%), and the majority of attempters were students 3 (27.3%) or homemakers 2 (18.2%). Suicidal behavior was mostly observed in unmarried people 6 (54.5%). Hanging was the most prevalent method (45.5%), and home (36.4%) and public places (36.4%) were equally the most frequent places of attempt. The consequential risk factors for the attempts were found to be marital problems/premarital affairs (50%), followed by unfulfilled demand/conflict (30%). While all 11 items depicted the method and place of the attempt, two also depicted the complete scene of the attempt. One item used language that normalized suicide as a constructive solution to the problem. None of the pictures publicized any mental health messages or educated the public about suicide prevention.

**Conclusions:**

The minimal adherence of the Nepali motion pictures on the depictions of suicidality with WHO media guidelines indicates urgent need to create awareness among the Nepali film fraternity.

## Abstract 2

This study assessed how suicidality has been depicted in Nepali movies (released between 1990‐2020) by scrutinizing their contents against the sociodemographic checklist and WHO media guidelines for suicidal reporting. Out of the scrutinized 573 movies, we found 10 movies, consisting of 11 characters showing suicidal behavior; while all 11 items mentioned the method and location of suicide, one used language which normalized suicide as a constructive solution to the problem. None of the pictures publicized any mental health messages or educated the public about suicide prevention. The minimal adherence to suicidal depiction indicated the urgent need to create awareness among the Nepali film fraternity.

## 1 INTRODUCTION

Suicide is a global public health priority incurring the loss of valuable lives worldwide, 77% of which occurred in low‐ and middle‐income countries(LMICs) in 2019 (World Health Organization, 2019). Nepal is also an LMIC where suicide is under‐prioritized as a public health issue. World Health Organization (WHO) estimated an age‐standardized suicide rate of 24.9 per 100,000 in Nepal, ranking it 7th globally (Marahatta et al., 2017). The burden of suicidal deaths in Nepal increased by 33% in 2019 compared to 2015, which was 30% (Silwal et al., 2021). Suicide reporting in the media is a potent environmental exposure that can influence suicide rates (Sisask & Värnik, 2012). The motion picture is one of the media that can contribute to fostering different behaviors depicted by the actors in the movies, as people, in general, tend to idolize or replicate the characters they resonate with (Till et al., 2015). The depiction of suicide in motion pictures has the potential to have an impact on suicidal conduct (Till et al., 2015), and there is abundant evidence that the media can have a substantial impact on either strengthening or weakening suicide prevention efforts (World Health Organization, 2023). Experimental research also explains that the sensational and explicit broadcasting of suicide depictions in motion pictures could increase the risk in vulnerable populations (Till et al., 2014). This assumption appears reasonable, given that mood management suggests that people choose media inputs that modify and regulate affective experiences and mood states in positive ways (World Health Organization, 2023; Till et al., 2014).

Despite the alarming level of suicide in Nepal, the lack of systematic reporting may underestimate public health needs or contribute to the misallocation of resources to groups most at risk (Hagaman et al., 2016). As suicide is against the law in Nepal, suicide deaths must be investigated, charged, and reported through the judicial system. Due to the government's lack of urgency in working with local stakeholders, there is no vital registration system in Nepal, which limits the system's ability to report suicides effectively and entrench WHO standard guidelines (Hagaman et al., 2016).

Motion pictures can play a significant role in fostering proper help‐seeking behaviors, especially among people with suicidal thoughts, in a country like Nepal, where mental health issues are rarely discussed in public due to stigma. In Nepal, 42 movies were released in 2022 (Pradhan et al., 2011), which signifies that motion pictures are commercial media and widely accepted. Reporting on suicidal behavior in the media and motion pictures in a responsible manner is one of the recognized public health strategies for preventing suicide (Rana, 2022). Research has highlighted the impact that media reporting has on the suicide rates in Nepal while also shown the inadequate receptiveness of World Health Organization (WHO) media reporting guidelines by the Nepalese media portals (printed and online) (Singh et al., 2022). However, motion pictures in Nepal are not into reflection while considering the content related to the suicidal portrayal. No detailed studies analyzing the depiction of suicide in Nepali movies and its impact on the general population have been found, which would be significant in considering population‐level prevention strategies. Therefore, we aimed to assess how suicidality has been depicted in Nepali movies by scrutinizing their contents against the sociodemographic checklist and WHO media guidelines for suicidal reporting. The findings of this study are expected to provide insights into how suicide is depicted in Nepali movies in accordance with WHO media guidelines and help to build awareness through population‐based strategies for suicide prevention.

## 2 METHOD

### 2.1 Data collection

This research is a narrative quantitative analysis of motion pictures containing suicidal behavior available in Nepali movies under the study objectives and instrument. We first searched on Google for the list of movies released in the past three decades (1990–2020). Then the movies were separated into a list and searched on YouTube (NP) (www.youtube.com), as it is publicly and freely accessible to all. The terms “suicide in Nepali movie” and “suicide in Nepali film” were used in Nepali and English to identify movies. The available movies were scrutinized to assess the sociodemographic variables and helpful and harmful characteristics of suicidal depiction as per the WHO media guidelines for suicidal reporting in movies. Three individual authors extracted the data independently, which were thoroughly checked by the lead author. However, the discrepancies between the raters were resolved by discussing them with the lead author. We included items from Nepali movies where suicide without any ambiguity on the nature of death, that is, suicide, homicide, or accidental, is shown. We excluded items where the attempts did not indicate suicide. We did not include motion pictures available in other than Nepali language.

### 2.2 Instruments

#### 2.2.1 Items for movie/drama

We noted the name of the movie/drama, release year, and type of the movie, such as romantic, social, dramatic, political, horror, and prominence of role/character (hero, heroine, or others cameo characters). In this study, “romantic movie” referred to movies with romantic love story with affectionate romantic involvement of the main characters, “social movies” referred to movies with more focus on depicting social issues, “dramatic movies” referred to movies featuring stories with high stakes and conflicts, portraying real‐life scenarios or extreme situations with emotionally driven characters, “political movies” referred to movies featuring stories related to political situations or politicians, and “horror movies” referred to movies featuring frightening and unnatural things happening.

#### 2.2.2 Sociodemography of the person with suicidal behavior

We extracted the sex of the index person, assumed age, educational attainment, occupation, religion, marital status, and socioeconomic status. For age estimation, we followed methods available in previously published paper (Trewavas et al., 2010).

#### 2.2.3 Questionnaire for suicidal behavior

We identified the type of suicidal behavior (fatal or nonfatal attempt), method of attempt, substance abuse before the attempt, any explicit risk factor, life events, place of suicide, depiction of the scene (complete/ incomplete), mental health message, relation to drug abuse, the social role of the person with suicidal behavior, suicide note, communication of thoughts, and educative material in the movie/drama.

#### 2.2.4 Quality of reporting

We assessed the movies and dramas against three positive and three negative characteristics of WHO media reporting guidelines (World Health Organization, 2017). Potentially positive characteristics included adequate information about help‐seeking for suicidal behavior, provision of facts and statistics on suicide, and supportive information regarding coping with life stressors or suicidal behavior as positive characteristics. On the other hand, the negative characteristics included language normalizing suicide or presenting it as a way out of a problem, description of methods, and details of the location of suicidal behavior as negative reporting characteristics. We decided on these six criteria based on previous studies (Arafat et al., 2022; Wang & Parris, 2021).

### 2.3 Statistical analysis

Data were analyzed descriptively using frequency and percentage in MS Excel (2010).

### 2.4 Ethical consideration

As the study analyzed publicly available information from the motion pictures, no formal ethical clearance was sought for conducting the study.

## 3 RESULTS

We scrutinized 573 movies, where we found ten movies depicting suicidal behavior, consisting of 11 characters showing suicidality, totaling the prevalence of suicidal behavior to 1.75%. Among the 11 characters, 8 (72.7%) were suicide, and 3 (27.3%) were suicide attempts. Of the 10 movies, one depicted two attempts (two cases: a suicide and an attempt), whereas the rest contained one item each. All the movies depicting suicidal issues were released after 2010, ranging from 2011 to 2020, because the movies before these decades did not contain any items that depicted suicidality. The name of the items, release year, link, the time of the scene, nature of the item, and type of the item have been provided in Supplementary File 1.

Among the 11 suicidal behavior, 8 (72.7%) were suicides, and the rest were attempts. The suicidal behavior was mostly seen in males 6 (54.5%). Most attempters were either students 3 (27.3%) or homemakers 2 (18.2%). Suicidal behavior was mostly observed in unmarried people 6 (54.5%). Majority of the characters that committed/attempted suicide were from lower socioeconomic class 4(36.4%). The detailed findings related to sociodemographic characteristics of people with suicidal behavior have been presented in Table 1.

**TABLE 1:** Sociodemographic characteristics of people with suicide attempts in Nepali movies (*n* = 10).

Characteristics	*n* (%)
Sex	
Male	6 (54.5%)
Female	5 (45.5%)
Age	
Middle adult	2 (18.2%)
Young adult	2 (18.2%)
Education	
Basic level	1 (9.1%)
University	2 (18.2%)
Occupation	
Student	3 (27.3%)
Homemaker	2 (18.2%)
Working abroad	1 (9.1%)
Marital status	
Unmarried	6 (54.5%)
Married	4 (36.4%)
Socioeconomic class	
Low	4 (36.4%)
Middle class	2 (18.2%)
Upper class	2 (18.2%)

The attempts (both fatal and nonfatal) were mainly depicted by the lead characters (hero/heroine), that is, 4 (36.4%), followed by 3 (27.3%) in cameo roles. Hanging was the most prevalent method 5 (45.5%), followed by drowning 2 (18.3%) and cutting the wrist 2 (18.3%). Inside home and outside of home (i.e., public places) were noted as the most frequent places of attempt 4 each (36.4% in home and 36.4% in public places), followed by bridges (18.2%). The detailed findings related to characteristics of the suicidal behavior have been shown in Table 2.

**TABLE 2:** Characteristics of suicidal behavior in Nepali movies (*n* = 10).

Characteristics	*n* (%)
Suicidal behavior	
Attempt	3 (27.3%)
Suicide	8 (72.7%)
Prominence of character (hero, heroine, or others)	
Lead actor(hero)	2 (18.2%)
Lead actress(heroine)	2 (18.2%)
Side actor	2 (18.2%)
Side actress	2 (18.2%)
Others (only in the scene)	3 (27.3%)
Method of attempt	
Hanging	5 (45.5%)
Poisoning	1 (9.1%)
Cutting in wrist	2 (18.2%)
Stabbing	1 (9.1%)
Drowning	2 (18.2%)
Place of suicide	
Bridge	2 (18.2%)
Home	4 (36.4%)
Public space	4 (36.4%)
Hostel	1 (9.1%)
Suicide notes	2 (18.2%)

The consequential risk factors for the attempts were found to be marital problems 4 (40%), followed by unfulfilled demand and conflict 3(30%) each, and premarital affair 2 (10%). The detailed risk factors of the suicidal behavior have been presented in Table 3.

**TABLE 3:** Risk factors for suicidal behavior in Nepali movies (*n* = 10).

Risk factor	*n* (%)
Defamation	1 (10%)
Extramarital affair	1 (10%)
Conflict	3 (30%)
Marital problems	4 (40%)
Mental illness	1 (10%)
Poverty	1 (10%)
Premarital affair	2 (20%)
Unemployment	1 (10%)
Unfulfilled demand	3 (30%)
Physical abuse by husband	1 (10%)

All 11 items (suicidal cases in the movies) mentioned the method and place of suicide attempts, whereas only 2 (18.2%) of the items depicted the complete scene of the suicide attempts. One item (9.1%) carried sensational language, which/normalized suicide and presented it as a constructive solution to the problem. None of the ten motion pictures publicized any mental health messages or educated the public about suicide prevention. The detailed quality assessment related to portraying of the suicidal behavior has been presented in Table 4.

**TABLE 4:** Quality assessment of reporting of suicide in movies and drama against WHO guidelines.

Characteristics	*n* (%)
Mentioning the methods	11 (100%)
Location of attempt	11 (100%)
Depiction of attempt	
Complete	2 (18.2%)
Partial	9 (81.8%)
Use language which sensationalizes or normalizes suicide or presents it as a constructive solution to problems	1 (9.1%)
Mental health message	0
Relation to drug abuse	0
Provide information about where to seek help	0
Educate the public about the facts of suicide and suicide prevention without spreading myths	0
Report stories of how to cope with life stressors or suicidal thoughts and how to get help	0

## 4 DISCUSSION

### 4.1 Major findings and interpretations

Our study shows that 36.4% of attempts (fatal, nonfatal) were observed in lead roles (heroes and heroines), and hanging was found as the most prominent method, followed by cutting the wrist and drowning. Both home and public places were identified as the commonest places of attempts 36.4% each, followed by a bridge (18.2%); males (54.5%) were slightly predominant as compared to females (45.4%) in the case of attempts (53.5%), 18.2% each of the suicidal behavior was noted in young adults and middle adults. However, the age of many characters was not able to be estimated. More attempts were found in unmarried people (54.5%), 27.3% of them were students. Premarital and extramarital affairs, sexual harassment, and unfulfilled demand (boy not marrying an unmarried pregnant girlfriend) were the most prominent risk factors. The potentially harmful characteristics (such as portraying of method and location of attempt, complete or partial depiction of attempt, and use language, which sensationalizes or normalizes suicide or presents it as a constructive solution to problems) were depicted clearly in all events, whereas potentially helpful contents (such as mentioning of mental health messages, provide information about where to seek help, educate the public about the facts of suicide and suicide prevention without spreading myths) were mentioned minimally.

A study conducted in Nepal shows similar results to our sociodemographic variables, where suicide cases have been reported more in males than females (Arafat et al., 2022). The majority of attempters were either students 3 (27.3%) or married males with lower socioeconomic conditions 4 (36.4%). A similar study in the United States illustrates that cinematic representations over the years have emphasized economic strains with increasing suicide rates. It is linked with different sociological rhetoric, which identified that movies highlighted the increasing suicide linked with socioeconomic factors like financial hardships and unemployment (demotions, adverse work conditions) comparatively more in men than in women (Pandey et al., 2022). However, the suicide attempts in our study were mostly seen in young adults, especially students. According to a study, those adolescents who experience symptoms of different mental health issues might find suicide as an acceptable solution to their problems influenced by suicide portrayals in fictional stories (Lester et al., 2014).

In this study, hanging was the most prevalent method (45.5%), followed by drowning and cutting the wrist. A similar finding was reported by another study, with hanging being predominantly found among the female population as a method of suicide while firearms in males (Jamieson & Romer, 2011). However, studies conducted in South Asian countries like India and Bangladesh highlight pesticide ingestion as a prominent method (Andriessen & Krysinska, 2020; Arafat et al., 2022; Jamieson & Romer, 2011; Lester et al., 2014; Pandey et al., 2022). As a developing and male‐dominant Nepalese society, we found that the male characters mostly attempted suicide due to financial hardships and the inability to improve their family's economic conditions. On the contrary, females attempted suicide due to extramarital affairs, premarital love affairs, and the breaking of commitments by partners in premarital affairs.

Studies conducted in Nepal have shown association between social risk factors (such as poverty, premarital affairs, and conflict) and suicide (Atteraya et al., 2021; Garrison‐Desany et al., 2020; Negi, 2017). These findings correspond with that of our study, where only 1 (10%) case was identified to have mental illness while others were due to conflict, premarital and marital issues, premarital pregnancy, and unfulfilled demand. These reasons, however, do not highlight that the reason for attempting suicide could be underlying mental illness. The National Mental Health Policy is not yet completely functional in Nepal, and no mental health act exists (Kohlbrenner et al., 2016); this could also be a reason for sensationalizing suicide as a solution to people's problems rather than the government strictly monitoring the contents being put forward to the public.

As mentioned in the WHO guidelines, the positive aspects were not highlighted in any events; instead, the potentially harmful of suicidal behaviors were discussed clearly. One item in our study showed the use of language sensational language which normalized suicide and presented it as a constructive solution to the problem. The stigma attached to mental illness in Nepali society, including the film fraternity here, may have acted as a key barrier and could also be a significant reason for presenting no constructive mental health messages to the public, instead depicting suicide as a panacea to their problems. Misleading statements about attempted suicide in any form of media reporting could lead to imprisonment, fines, or both for those who attempt suicide, as has been stated in Nepalese publications on the issue of suicide (Marahatta et al., 2017). This can act as a source of discouragement for the stakeholders of the film fraternity to convey messages of suicide‐related awareness and the helplines available in Nepal through motion pictures. Additionally a study conducted in Nepal on the quality of media reporting in newspapers also showed similar results of negligible useful reporting traits that the WHO guideline encourages to follow (Singh et al., 2022). These similar depictions and the portrayal of negative aspects, despite the variation of suicidal behavior in the movies, could act as a conduit to influence the general public.

### 4.2 Implications

The study indicates a lower presence of suicidality in motion pictures in Nepal. Also, it indicates an extreme lack of awareness of the impact of depicting suicidality in movies on the community and available guidelines for media reporting of suicidal behavior in motion pictures. With the minimal adherence of the suicidal depiction to WHO guidelines, as shown in our data, creating awareness among the film fraternity, including the directors, scriptwriters, actors, producers, and cinematographers, is pivotal. A joint venture of government, media, and mental health professionals could entrench awareness and help build up training materials. A movie assessment highlighting the helpful characteristics must be thoroughly reviewed with mental health professionals before release. This could help establish motion pictures as a profound platform to spread positive mental health messages, reduce glorifying suicide as the only solution, and increase pragmatic approaches to protective actions against suicide among a mass audience.

### 4.3 Strengths

This study is an attempt to assess the depiction of suicidal behavior in Nepali motion pictures, perhaps the first study in Nepal. This research will be conducive to the film fraternity as well as upcoming researchers as a benchmark to understanding and encourage how sensible media reporting could hence lead to a suicide prevention strategy.

### 4.4 Limitations

In order to generalize the results of the present study, we established some limitations. We have included only Nepali motion pictures publicly available on “*YouTube*,” because we could not assess motion pictures from other paid platforms. As a diverse nation with numerous languages spoken all over the country, we did not include any motion pictures released in other local languages. This may compromise the inclusivity of the article. There is no potent evidence of imitative acts of suicide in populations exposed to suicide in entertainment media (Cousins, 2016; Ferguson, 2019). The sample size is relatively small, restricting the generalization of the results. The sociodemographic variables were extracted based on the subjective assessment. We did not extract of the variables due to the nature of data (age or marital status, or education of the characters were not mentioned in the motion picture). We noted the prominent risk factors in most of the target variables/suicide attempters, however, it is important to note that suicide is multifactorial and there might be some interaction with other different risk factors. We did not assess the representativeness of the sample due to the nature of the data

## 5 CONCLUSIONS

This study shows the negligible interest of the Nepali movie fraternity in sensitizing people to the potentially helpful characteristics of suicide prevention. With the increasing number of movies released over the past decade, future studies must include movies and dramas from other paid platforms to nudge deeper into the complexity of suicidal behavior. It will help to accumulate comprehensive data on the variations of suicidal behavior and enhance the standard of media reporting according to the WHO guidelines.

## AUTHOR CONTRIBUTIONS

**Rakesh Singh**: Conceptualization; data curation; formal analysis; investigation; methodology; project administration; resources; software; supervision; validation; visualization; writing—original draft; writing—review and editing. **Sharika Mahato**: Conceptualization; data curation; formal analysis; investigation; methodology; project administration; resources; software; supervision; validation; visualization; writing—original draft; writing—review and editing. **Kritika Singh**: Formal analysis; investigation; project administration; writing—original draft; writing—review and editing. **Niranjan Raman Puri**: Investigation; project administration; Writing—review and editing. **Susma Baniya**: Investigation; project administration; writing—review and editing. **S. M. Yasir Arafat**: Conceptualization; writing—review and editing.

## FUNDING

The authors received no financial support for this article's research, authorship, and publication.

## CONFLICT OF INTEREST STATEMENT

All other authors declared no potential conflicts of interest concerning this article's research, authorship, and publication.

## TRANSPARENCY STATEMENT

The lead author Rakesh Singh reported that this manuscript is an honest, accurate, and transparent account of the study being reported, that no important aspects of the study have been omitted, and that any discrepancies from the study as planned have been explained.

### PEER REVIEW

The peer review history for this article is available at https://publons.com/publon/10.1002/brb3.3366.

## Supporting information

Supporting Information

## Data Availability

The data supporting this study's findings are available from the corresponding author upon reasonable request.
