# Supplementary material for: Suicidal Behavior in Nepali motion pictures of three decades (1990–2020): A content analysis
Source: Brain Behav. 2023 Dec 31;14(1):e3366. doi: 10.1002/brb3.3366 (PMC10757900; doi:10.1002/brb3.3366)
Supplement: Supplementary file 1 — Supporting Information [file BRB3-14-e3366-s001.docx]

Supplementary File 1: List of Nepali movies depicting suicidal behavior

| S.N. | Title of Movie | Year | YouTube Weblink | Time stamp | Type of movie |
| --- | --- | --- | --- | --- | --- |
| 1 | Doli | 2018 | <https://www.youtube.com/watch?v=_hOWKKdPIvU&t=4411s> | 1:13:57; 1:07:24 | Social |
| 2 | Kagaz Patra | 2019 | <https://www.youtube.com/watch?v=3UF92drF0n4> | 1:30:41 | Romantic Comedy, Social |
| 3 | Saili |  | <https://www.youtube.com/watch?v=p8_YI_dNImM> | 2:01:58 | Romantic, Social |
| 4 | Gangster blues | 2017 | <https://www.youtube.com/watch?v=m1CQGrg2Z_o> | 2:05:34 | Action, Romantic, Crime |
| 5 | Apabad | 2012 | <https://www.youtube.com/watch?v=1oIh8IFv-ms> | 6:24,11:34 | Social, Drama |
| 6 | Hostel Returns | 2016 | <https://www.youtube.com/watch?v=HANVpfzOFUE> | 1:53:46 | Romantic |
| 7 | Sunkeshari | 2018 | <https://www.youtube.com/watch?v=Cz29r8qm07k&t=335s&ab_channel=BGEntertainment> | 5:37 | Horror |
| 8 | Ekpal | 2016 | <https://www.youtube.com/watch?v=FT88lOhd3Pk> | 1:46:14 | Romantic |
| 9 | Jhumkee | 2016 | <https://www.youtube.com/watch?v=-v9XddzDkLc> | 1:13:27 | Romantic, Social |
| 10 | Jeevan Mrityu | 2008 | <http://www.youtube.com/HiTechEntertai>.. | 1:34:59 | Romantic |
